# Interfacially Locked Metal Aerogel Inside Porous Polymer Composite for Sensitive and Durable Flexible Piezoresistive Sensors

**DOI:** 10.1002/advs.202201912

**Published:** 2022-06-24

**Authors:** Jian Li, Ning Li, Yuanyuan Zheng, Dongyang Lou, Yue Jiang, Jiaxi Jiang, Qunhui Xu, Jing Yang, Yujing Sun, Chuxuan Pan, Jianlan Wang, Zhengchun Peng, Zhikun Zheng, Wei Liu

**Affiliations:** ^1^ The Key Laboratory of Low‐Carbon Chemistry & Energy Conservation of Guangdong Province Key Laboratory for Polymeric Composite and Functional Materials of Ministry of Education State Key Laboratory of Optoelectronic Materials and Technologies School of Materials Science and Engineering Sun Yat‐sen University Guangzhou 510006 P. R. China; ^2^ Key Laboratory of Optoelectronic Devices and Systems of Ministry of Education and Guangdong Province College of Physics and Optoelectronic Engineering Shenzhen University Shenzhen 518060 P. R. China; ^3^ Center for Advanced Mechanics and Materials Applied Mechanics Laboratory Department of Engineering Mechanics Tsinghua University Beijing 100084 P. R. China; ^4^ Key Laboratory for Polymeric Composite and Functional Materials of Ministry of Education State Key Laboratory of Optoelectronic Materials and Technologies School of chemistry Sun Yat‐sen University Guangzhou 510006 P. R. China

**Keywords:** interface, metal aerogels, piezoresistive sensors, polymer, porous materials, pressure sensors

## Abstract

Flexible pressure sensors play significant roles in wearable devices, electronic skins, and human‐machine interface (HMI). However, it remains challenging to develop flexible piezoresistive sensors with outstanding comprehensive performances, especially with excellent long‐term durability. Herein, a facile “interfacial locking strategy” has been developed to fabricate metal aerogel‐based pressure sensors with excellent sensitivity and prominent stability. The strategy broke the bottleneck of the intrinsically poor mechanical properties of metal aerogels by grafting them on highly elastic melamine sponge with the help of a thin polydimethylsiloxane (PDMS) layer as the interface‐reinforcing media. The hierarchically porous conductive structure of the ensemble offered the as‐prepared flexible piezoresistive sensor with a sensitivity as high as 12 kPa^−1^, a response time as fast as 85 ms, and a prominent durability over 23 000 compression cycles. The excellent comprehensive performance enables the successful application of the flexible piezoresistive sensor as two‐dimensional (2D) array device as well as three‐dimensional (3D) force‐detecting device for real‐time monitoring of HMI activities.

## Graphical abstract

An “interfacial locking strategy” is developed to design metal aerogel‐based flexible piezoresistive sensors with both high sensitivity and prominent durability. The interpenetrated interface‐reinforcing PDMS layer tightly grafts the metal aerogels on the highly elastic and mechanically stable sponge, breaking the bottleneck of intrinsically poor mechanical strength of metal aerogels, and significantly expanding their applications in emerging fields.

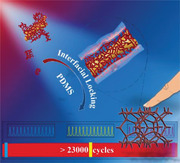

## 1 Introduction

High‐performance flexible piezoresistive pressure sensors play significant roles in various areas, such as wearable devices, human‐machine interfaces, electronic skins, and Internet of Things.^[^ ^1^ ^]^ Unfortunately, it remains challenging to develop flexible piezoresistive pressure sensors with outstanding comprehensive performances, especially excellent long‐term durability and repeatability that is critical from practical application point of view.

Among various materials for flexible piezoresistive pressure sensors, 3D porous materials with intrinsically conductive backbones (3DPMICB) (such as neat conductive sponge, conductive materials coated sponge, etc.) have attracted great attention.^[^ ^2^ ^]^ On the other hand, 3DPMICB often suffer from insufficient stability and durability due to irreversible damage during compression, even with the help of polymers.^[^ ^2c‐e^ ^]^ Alternative strategy of dipping or coating conductive materials (such as metal particles,^[^ ^3^ ^]^ metal nanowires,^[^ ^4^ ^]^ carbon nanotubes,^[^ ^5^ ^]^ graphene,^[^ ^6^ ^]^ and MXene^[^ ^7^ ^]^) on the insulating porous elastic matrixes (such as Thermoplastic polyurethanes (TPU), polyurethane (PU), and porous PDMS) has been developed to fabricate flexible piezoresistive pressure sensor.^[^ ^4^, ^8^ ^]^ Generally, flexible pressure sensors based on porous elastic substrates with excellent mechanical stability are supposed to have good durability.^[^ ^9^ ^]^ However, the strength of the interfacial interaction seriously affects the performance of the flexible sensor.^[^ ^10^ ^]^ Due to the weak physical interaction and modulus mismatch between the conductive material and the porous elastic substrate, when subjected to long‐term cyclic compressive strain, the conductive material easily aggregates and slides, or even falls off from the matrix, resulting in unstable sensor signal.^[^ ^11^ ^]^ For example, Lu and co‐workers fabricated a promising flexible pressure sensor that could withstand 50 000 cycles by coating carbon black on the sponge through layer‐by‐layer assembly, but the cycle curve has a relatively large drift and fluctuation.^[^ ^12^ ^]^ To enhance the stability and repeatability of such flexible pressure sensor, the key is to construct good interfacial interaction between the conductive material and the elastic substrate.^[^ ^2^, ^10^, ^11^, ^13^ ^]^ Although Fu and co‐workers used oxygen plasma to modify the surface of PU foam to enhance the interfacial interaction between the conductive material and the framework and obtained high stability, the cyclic curve still has large fluctuations.^[^ ^14^ ^]^ Therefore, it urgently calls for effective strategies to prepare flexible pressure sensors with excellent comprehensive performances, in particular outstanding durability and repeatability.

As an emerging class of 3DPMICB developed in recent years,^[^ ^15^ ^]^ metal aerogels have shown promising applications in a variety of areas,^[^ ^16^ ^]^ especially in electrocatalysis,^[^ ^17^ ^]^ due to their unique characteristics of large specific surface area, high porosity, and conductive interconnected nanowire network structure.^[^ ^17^, ^18^ ^]^ These special characteristics are also very beneficial for sensing pressure, however, metal aerogels have few chance to stand and show on the flexible pressure sensor stage, mainly limited by their poor mechanical property.^[^ ^15a^ ^]^ Despite a pressure sensor assembled by Cu nanowire aerogel showing 80 ms response time and moderate durability at 30% compressive strain for 200 cycles,^[^ ^2c^ ^]^ in most cases, the 3D intrinsically conductive porous nanowire network of metal aerogels (especially those assembled from metal nanoparticles) are easily destroyed when deformed.^[^ ^15a^ ^]^ Recently, we have developed a silicone‐confined gelation strategy to integrate metal aerogels with macro‐porous skeletons and ensure metal aerogels to preserve their intrinsic structure improving the durability of metal aerogels.^[^ ^19^ ^]^ However, metal aerogels in such integrated hierarchical porous structure is still prone to be irreversibly deformed and damaged under pressure. It is highly demanded while challenging to develop strategies to bridge the gap between metal aerogels and flexible piezoresistive pressure sensor.

Herein, we propose a facile “interfacial locking strategy” to construct metal aerogels based flexible pressure sensors with excellent comprehensive performance including both high sensitivity and prominent durability. Ag_2_Au_3_ alloyed bimetallic aerogel (2, 3 represent the volume ratio of concentrated Ag and Au nanoparticles solution during preparation) was chosen as the core sensing material to fabricate the metal aerogel based flexible pressure sensor, due to its easy integration with flexible sponge via silicone‐confined gelation strategy, and its good electrical conductivity, chemical stability, and biocompatibility. More importantly, even though the mechanical strength of the Ag_2_Au_3_ aerogel is poor, it is not as fragile as metal aerogels of other metal components, such as Pt, Pd, and PtPd^[^ ^15^, ^18^, ^19^ ^]^ (Video S1, Supporting Information), it has low modulus and can undergo plastic deformation^[^ ^20^ ^]^ (Video S1, Supporting Information), which makes the Ag_2_Au_3_ aerogel being able to better adapt to the deformation of the melamine sponge during the compression processes. The Ag_2_Au_3_ metal aerogel is perfectly grafted on the elastic melamine sponge support with the help of an appropriate binding layer of PDMS, forming a highly elastic and hierarchically porous structure. The melamine sponge provides elasticity for the flexible pressure sensors and the 3D porous Ag_2_Au_3_ aerogel combined with the porous sponge helps improve the sensitivity of flexible pressure sensors. The thin PDMS layer interpenetrated and tightly adhered to the Ag_2_Au_3_ aerogel surface makes the flexible pressure sensor robust, achieving long‐term stability over 23 000 cycles. Moreover, the flexible pressure sensor has a high sensitivity of 12 kPa^−1^, 84 ms response time, and 80 ms recovery time. The flexible pressure sensor shows excellent performances in monitoring human activities, and can also be fabricated into arrays devices and 3D force devices with good performance.

## 2 Results and Discussion

### 2.1 Preparation and Characterization

**Figure 1:**
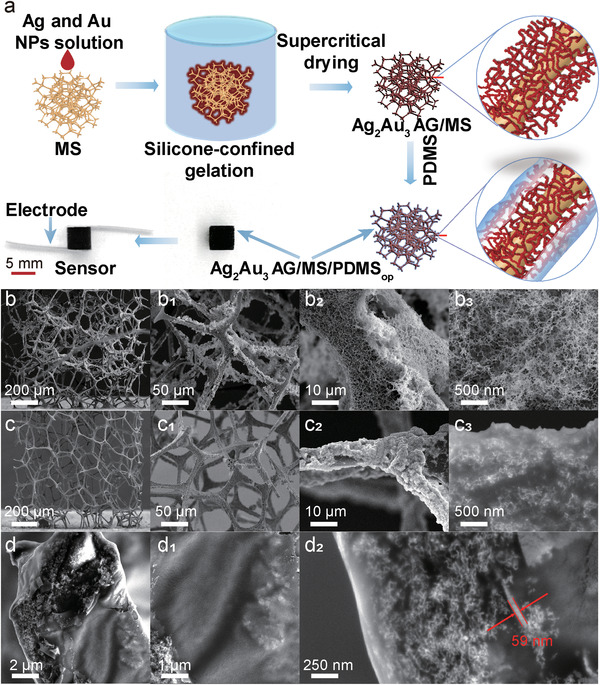
Preparation process and characterizations of the Ag_2_Au_3_ AG/MS/PDMS_op_ flexible sensor. a) Schematic illustration for the preparation of Ag_2_Au_3_ AG/MS/PDMS_op_ sensing layer and flexible pressure sensor. SEM images of b–b_3_) Ag_2_Au_3_ AG/MS, c–c_3_) Ag_2_Au_3_ AG/MS/PDMS_op_, SEM images of d–d_2_) the tentatively destroyed sites of Ag_2_Au_3_ AG/MS/PDMS_op_.

The fabrication of the flexible pressure sensor consists of two steps: the preparation of the Ag_2_Au_3_ AG/MS/PDMS_op_ (AG is aerogel, MS represents melamine sponge, “op” represents the optimized mass of PDMS (0.07 g) dissolved in 30 mL of *n*‐hexane solution) sensing layer and the fabrication of the device. As shown in **Figure** **1**a, for the preparation of the Ag_2_Au_3_ AG/MS/PDMS_op_ sensing material, the preprepared concentrated Ag and Au nanoparticle solutions were first injected into the melamine sponge. The resulting sponge was then soaked in silicone oil and heated to 75 °C to form Ag_2_Au_3_ hydrogel supported on MS. The Ag_2_Au_3_ AG/MS was obtained after supercritical drying. Finally, the Ag_2_Au_3_ AG/MS was immersed in a *n*‐hexane solution containing unpolymerized PDMS with low concentration, taken out, and heated to 75 °C for polymerization to obtain the Ag_2_Au_3_ AG/MS/PDMS_op_. The flexible pressure sensor is fabricated by installing copper foil electrodes (pasted on the polyimide film) on the upper and lower sides of the Ag_2_Au_3_ AG/MS/PDMS_op_ sensing material. (Figure S1a, Supporting Information).

**Figure 2:**
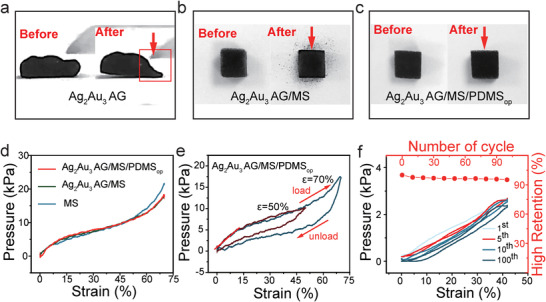
Mechanical properties of the Ag_2_Au_3_ AG/MS/PDMS_op_ flexible pressure sensor. Photographs of the a) Ag_2_Au_3_ AG before and after being pressed. The photographs of b) Ag_2_Au_3_ AG/MS and c) Ag_2_Au_3_ AG/MS/PDMS_op_ before compression and after 10 cycles of compression at 80% strain. Compressive stress–strain curves of d) MS, Ag_2_Au_3_ AG/MS, Ag_2_Au_3_ AG/MS/PDMS_op_. e) Compressive stress–strain curves of Ag_2_Au_3_ AG/MS/PDMS_op_ with 50–70% strain. f) Compressive stress–strain curves and high retention of Ag_2_Au_3_ AG/MS/PDMS_op_ with different cycles.

To obtain good elastic performance, a melamine sponge with pore size of 30–130 µm and polymer skeletons of ≈10 µm in width (Figure S1b, Supporting Information) was chosen as a matrix. The sponge displays good compression performance (**Figure** **2**d). For comparison with the Ag_2_Au_3_ AG/MS/PDMS_op_, Ag_2_Au_3_ AG and Ag_2_Au_3_ AG/MS were also prepared, and their compositions, morphologies, and structures were discussed in detail. Typically, Ag_2_Au_3_ AG is black lumpy solids (Figure S1c, Supporting Information). The transmission electron microscopy (TEM) image (Figure S1d, Supporting Information) and the scanning electron microscopy (SEM) images (Figure S1e–e_3_, Supporting Information) of Ag_2_Au_3_ AG show that lots of thin metal nanowire ligaments (≈20 nm in diameter) interconnect with each other to form a 3D porous conductive network. The Ag/Au atomic ratio of Ag_2_Au_3_ AG determined by TEM energy‐dispersive X‐ray spectroscopy (TEM‐EDS) is ≈51.5/48.5 (Figure S1f, Supporting Information). After the metal aerogels were grafted on the white melamine sponge, the melamine sponge changed from white to black (Figure 2b; Figure S1b_1_, Supporting Information). As shown from the SEM images of Ag_2_Au_3_ AG/MS in Figure 1b–b_3_, the Ag_2_Au_3_ AG intertwines on the 3D skeletons of the melamine sponge with their intrinsic morphology well preserved. In the case of the Ag_2_Au_3_ AG/MS/PDMS_op_, as shown in Figure 1c–c_3_ and Figure 1d–d_2_, it has a hierarchical structure with the sponge skeleton as the matrix, the metal aerogel as the intermediate sensing layer, and the PDMS membrane as the protecting layer (≈59 nm). The PDMS interpenetrates and wraps the surface part of the Ag_2_Au_3_ AG and tightly locks the Ag_2_Au_3_ AG and the melamine sponge skeleton together. The introduction of PDMS causes a certain extent of pore shrinking on the surface layer of the Ag_2_Au_3_ AG, while the intrinsic structures of Ag_2_Au_3_ AG is mostly conserved below the PDMS layer (Figure 1c_3_ and d_2_). As shown in the SEM‐EDS mapping results of Ag_2_Au_3_ AG/MS/PDMS_op_ in Figure S2a (Supporting Information), in the areas where there are more silver and gold elements, the content of silicon is also higher. These results further indicate the penetration and polymerization of PDMS inside the pores of Ag_2_Au_3_ AG (mainly on the surface part) and accumulation of more PDMS than that on the melamine sponge skeleton.

According to the attenuated total reflectance infrared (ATR‐IR) spectroscopy (Figure S2b, Supporting Information), the deformation vibration absorption peak of the triazine ring in melamine appears at 812 cm^−1^, and the aromatic ‐C=N stretching vibration peak of the triazine ring appears at 1540 cm^−1^, which can be attributed to the melamine sponge.^[^ ^21^ ^]^ When the metal aerogels grow on the melamine sponge, there is basically no change in the infrared spectrum. After the introduction of PDMS layer, three groups of peaks appear at 1260, 1017, and 797 cm^−1^ corresponding to ‐CH_3_ deformation vibration, symmetrical Si‐O‐Si stretching, and Si‐C rocking peaks, respectively, suggesting the existence of PDMS.^[^ ^22^ ^]^ From the X‐ray diffraction (XRD) (Figure S2c, Supporting Information), compared with the melamine sponge, the Ag_2_Au_3_ AG/MS shows four dominant peaks at 38.2°, 44.4°, 64.6°, and 77.6°, corresponding to a typical face‐centered cubic structure. Compared with the cubic Ag phase (JCPDS‐04‐0783), the XRD patterns of the Ag_2_Au_3_ AG/MS exhibit a slight positive shift owing to the formation of AuAg alloy.^[^ ^19^, ^23^ ^]^

In short, these above‐mentioned characterizations demonstrate that Ag_2_Au_3_ AG has been successfully grafted on the melamine sponge with the help of a thin PDMS interfacial locking layer, forming Ag_2_Au_3_ AG/MS/PDMS_op_ with hierarchically porous structure.

### 2.2 Mechanical Properties

As for the Ag_2_Au_3_ AG used here, it is susceptible to plastic deformation under pressure, as shown in Figure 2a and Video S1 (Supporting Information). Once light pressure is applied to the Ag_2_Au_3_ AG, it will deform easily and cannot rebound when the pressure is removed, which reveals the low modulus and certain plasticity of Ag_2_Au_3_ AG. These properties on one hand make the Ag_2_Au_3_ AG being able to better adapt to the deformation of the melamine sponge during the compression processes. On the other hand, it is necessary to select highly elastic porous melamine sponge to support the Ag_2_Au_3_ AG. However, as shown in Figure 2b, the Ag_2_Au_3_ AG/MS obtained by growing the Ag_2_Au_3_ AG on the melamine sponge can still not withstand cyclic compression at 80% strain. Black Ag_2_Au_3_ AG powder can be observed to fall off the sponge after this process. As shown in Figure S3a,b (Supporting Information), the metal aerogel fell from the melamine sponge skeleton, while the melamine sponge skeleton did not change significantly. This indicates that the interfacial force between the Ag_2_Au_3_ AG and the sponge skeleton is not strong enough.

Thus, an “interfacial locking strategy” of using a protecting layer of PDMS to make the Ag_2_Au_3_ AG/MS firmer is developed. Figure 2c show the strong reinforcement effect of PDMS on the composite material. There is no more Ag_2_Au_3_ AG falling even after repeated pressing. The morphology and structure did not show obvious change, as shown in Figure S3c,d (Supporting Information). The compression stress–strain curves of the MS, Ag_2_Au_3_ AG/MS, and Ag_2_Au_3_ AG/MS/PDMS_op_ show little difference, as shown in Figure 2d. These results prove that the introduction of Ag_2_Au_3_ AG and PDMS had little effect on the modulus of the melamine sponge, which can be attributed to the low modulus of the Ag_2_Au_3_ AG and the low amount of PDMS.

In addition, as shown in Figure 2e, Ag_2_Au_3_ AG/MS/PDMS_op_ exhibits excellent elasticity. When the applied strain is 50% and 70%, the hysteresis loops display similar shapes and become steeper with the increasing strain. The loading curve is characterized by the linear elastic area of the sponge skeleton bending, then the platform area of buckling phenomenon, and then the densification area that is attributed to the closure of the sponge pores.^[^ ^24^ ^]^ Notably, the strength of the Ag_2_Au_3_ AG/MS/PDMS_op_ sensing material is great enough to support repeated compression, in contrast to the Ag_2_Au_3_ AG. As shown in Figure 2f, the fifth hysteresis loop of the Ag_2_Au_3_ AG/MS/PDMS_op_ shrinks compared to the first curve, but the maximum stress at *ε* = 41% shows only a slight decrease, and more importantly it remains largely stable in the later cycles, which is often observed in sponge materials.^[^ ^11a^ ^]^ In addition, the hysteresis loop does not change significantly in the later cycles, indicating that no substantial internal structural changes occurred during the 100 cycles. Furthermore, the height of the Ag_2_Au_3_ AG/MS/PDMS_op_ sponge drops to 97.7% in the first 10 compression cycles. In the subsequent cyclic compression, the height decreases slightly but still maintains 95.2% of the initial height after 100 repeated compressions, indicating that the Ag_2_Au_3_ AG/MS/PDMS_op_ has good resilience. The stable 3D hierarchically porous structure, low density, high elasticity, and strong mechanical strength of Ag_2_Au_3_ AG/MS/PDMS_op_ make it a promising candidate material for pressure sensing.

### 2.3 Sensing Performance of the Ag_2_Au_3_ AG/MS/PDMS_op_ Based Flexible Pressure Sensor

**Figure 3:**
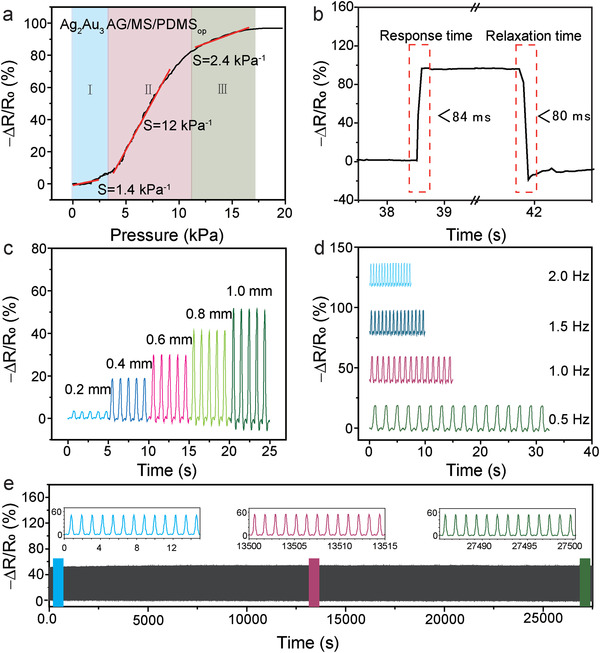
Sensing performance of the Ag_2_Au_3_ AG/MS/PDMS_op_ flexible pressure sensor. a) Typical sensitivity curve of the Ag_2_Au_3_ AG/MS/PDMS_op_ flexible pressure sensor. b) Response and relaxation time of the pressure sensor under rapid loading–unloading. c) −∆*R*/*R*_0_ response at different compression displacements. d) −∆*R*/*R*_0_ response at different compression frequencies. e) The response of the sensor was compressed over 23 000 cycles at a strain of 10% and a frequency of ≈0.9 Hz.

The sensing performance of Ag_2_Au_3_ AG/MS/PDMS_op_ based flexible pressure sensor is presented in **Figure** **3**. In general, the flexible piezoresistive pressure sensor needs to keep the resistance stable under different operating conditions, such as different voltages and pressures. The current versus voltage (*I*–*V*) curves (Figure S4a, Supporting Information) of the Ag_2_Au_3_ AG/MS/PDMS_op_ pressure sensor show good linear relationship in the voltage from −1 to 1 V under different pressures from 0 to 12.0 kPa, implying that composite sponge and the electrode are in good Ohmic contact. The slope of the *I*–*V* curves increases with the increase of pressure, illustrating that the resistance of the sensor decreases with the increasing pressure. The sensitivity (*S*) of the flexible pressure sensor can be calculated by S = −(∆*R*/*R*_0_)/∆*P*, where ∆*R* is the resistance change under different pressures *P*, and *R*_0_ is the initial resistance without pressure. As shown in Figure 3a, the (*R_0_−R*)/*R*_0_‐Pressure curve of the Ag_2_Au_3_ AG/MS/PDMS_op_ based flexible pressure sensor exhibits three different sensitivity regions. At low pressure region from 0 to 3.0 kPa (region I), the sensitivity is ≈1.4 kPa^−1^. When the pressure is further increased to 12.0 kPa (region II), the sensitivity quickly increases to 12.0 kPa^−1^. Above 12.0 kPa (region III), the sensitivity reduces to 2.4 kPa^−1^. The macroscopic compression process of the sensor is schematically shown in Figure S4d (Supporting Information). Under the small pressure in region I, the sponge skeleton bends, so that only the metal aerogels in the bending parts of the skeleton deforms to form new conductive channels, leading to lower extent of resistance decrease.^[^ ^25^ ^]^ With the increase of pressure to region II, the bending angle of many sponge skeletons increases, making the skeletons rotate,^[^ ^26^ ^]^ contact, and squeeze with each other until the pores are almost closed. During these processes, more and more pores of the internal metal aerogels are closed, forming more conductive channels and resulting in a rapid drop in resistance and correspondingly higher sensitivity.^[^ ^23^, ^26^ ^]^ In the very high pressure region III, the left sponge pores that could not be contacted in the previous stages are further closed, thereby closing the remaining metal aerogels voids, leading to further decrease of resistance but not as obvious as that in region II.^[^ ^27^ ^]^ From a microscopic perspective, the bending, contact and extrusion of the sponge skeletons all lead to the extrusion of a large number of metal aerogels at the corresponding positions, and the pores of the metal aerogels are closed to form more conductive channels, resulting in a decrease in electrical resistance, as shown in Figure S4e (Supporting Information). The detection limit is 0.252 kPa (Figure S4b, Supporting Information), that means it can monitor the touch and press of our fingers. Usually, when flexible pressure sensors are used for human motion monitoring, the flexible pressure sensors need to be able to respond to external stimuli in real time, at least lower than the human body's reaction time (<100 ms).^[^ ^28^ ^]^ The resistance of the Ag_2_Au_3_ AG/MS/PDMS_op_ flexible pressure sensor decreases rapidly when pressed rapidly, and recover to the original value after pressure removal (Figure 3b). The response time and the relaxation time of the Ag_2_Au_3_ AG/MS/PDMS_op_ flexible pressure sensors are shorter than 84 and 80 ms, respectively, indicating acceptable response/relaxation speed. The hysteresis of the response time and the relaxation time of this pressure sensor may be attributed to the short relaxation time of the melamine sponge.^[^ ^1b^ ^]^

What is more important, due to the support of elastic sponge and the interlocking of ultra‐thin elastic PDMS layer, the flexible pressure sensor exhibits good dynamic stability. As shown in Figure 3c, it can be seen from the curve that the heights of the first cycle curve and the fifth cycle curve are basically the same, and the height of the curve increases with the increase of the compression displacement, even after a quick shock, the resistance remains stable (Figure S4c, Supporting Information), indicating its sensitivity to the compression displacement and good dynamic stability. As shown in Figure 3d, the −Δ*R*/*R*_0_ profile has no obvious compression frequency dependence within the frequency range from 0.5 to 2.0 Hz, demonstrating the flexible pressure sensor is sensitive and stable at different compression frequencies. This means that the sensor can respond well to high and low compression frequencies and different compression displacement.

Moreover, durability is a very important parameter for the flexible pressure sensor. The Ag_2_Au_3_ AG/MS/PDMS_op_ flexible pressure sensor shows a highly stable response even after >23 000 cycles of loading and unloading of pressure (Figure 3e). The insets of Figure 3e show the 13 cycles of the resistance response at the inception (left), intermediation (middle), and termination (right) of the stability test. The response is very stable, and no obviously drift of resistance has been observed, which can be attributed to the reinforcement of metal aerogels on the sponge skeletons by the thin and flexible PDMS interface locking layer.

The comprehensive performance especially the stability of the Ag_2_Au_3_ AG/MS/PDMS_op_ piezoresistive flexible pressure sensor is among the top when compared with those metal based ones reported in literature (Table S1, Supporting Information). Even when compared with those based on other sensing materials,^[^ ^2^, ^8^ ^]^ the Ag_2_Au_3_ AG/MS/PDMS_op_ flexible pressure sensor can distinguish itself as well.

### 2.4 Influence Parameters

**Figure 4:**
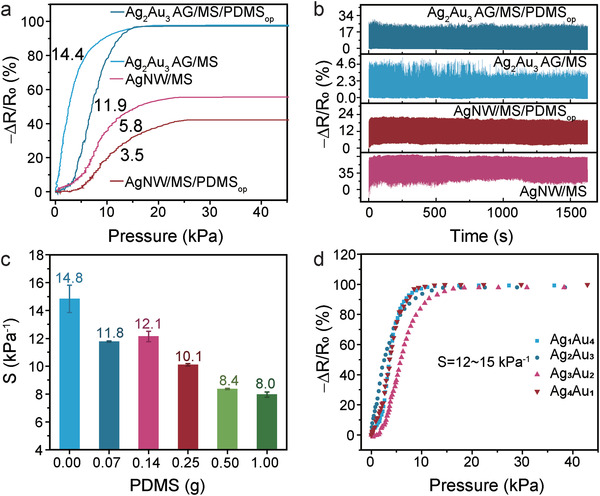
Influencing factors. a) Sensitivity of the Ag_2_Au_3_ AG/MS, Ag_2_Au_3_ AG/MS/PDMS_op_, Ag NW/MS, and Ag NW/MS/PDMS_op_ flexible pressure sensors. b) Cycling stability of Ag_2_Au_3_ AG/MS, Ag_2_Au_3_ AG/MS/PDMS_op_, Ag NW/MS, and Ag NW/MS/PDMS_op_ through 2000 compression cycles at 10% strain and ≈1 Hz. c) Effect of PDMS content on maximum sensitivity. d) The (*R_0_*−*R*)/*R*_0_‐Pressure curves of the Ag_X_Au_Y_ AG/MS with different silver and gold ratio.

The influences of various factors on the sensing performance are systematically investigated. First, Ag_2_Au_3_ AG/MS based and Ag_2_Au_3_ AG/MS/PDMS_op_ based flexible pressure sensor were prepared to study the influence of PDMS on the sensitivity and stability of flexible pressure sensor. As shown in **Figure** **4**a, the sensitivity of the Ag_2_Au_3_ AG/MS/PDMS_op_ based flexible pressure sensor (11.9 kPa^−1^) is lower than that of the Ag_2_Au_3_ AG/MS based flexible pressure sensor (14.4 kPa^−1^), indicating that the introduction of PDMS protecting layer in the flexible pressure sensor reduces the sensitivity. Despite this, the use of PDMS greatly enhances the stability of the flexible pressure sensor. As shown in Figure 4b, the 2000‐cycle curves of the Ag_2_Au_3_ AG/MS flexible pressure sensor without PDMS show obvious drift and float. However, the ensemble of 2000‐cycle curves of the Ag_2_Au_3_ AG/MS/PDMS flexible pressure sensor is much smoother and much less drifting, which is mainly attributed to the interfacial reinforcement of the Ag_2_Au_3_ AG and the sponge skeleton by the PDMS. The Ag_2_Au_3_ AG/MS/PDMS_op_ is strong enough to stand the continuous loading and unloading process. For comparison, flexible pressure sensor based on silver nanowires dip‐coated on the melamine sponge (Ag NW/MS, Figure S5a, Supporting Information) are also fabricated. After coating with PDMS (Figure S5b, Supporting Information), the changes in sensitivity and stability are the same as those for the Ag_2_Au_3_ AG/MS based flexible pressure sensor. The sensitivity is reduced from 5.8 kPa^−1^ for the Ag NW/MS/PDMS sensor to 3.5 kPa^−1^ for the Ag NW/MS sensor, while the stability is improved, as shown in Figure 4a,b. Furthermore, increasing the content of PDMS can reduce the influence of environmental humidity on the flexible pressure sensors (Figure S6, Supporting Information). However, excessive use of PDMS will quickly reduce the sensitivity of the flexible sensor. As shown in Figure 4c, increasing the concentration of PDMS from 0 to 1 g makes the sensitivity of flexible pressure sensor drop from 14.8 to 8.0 kPa^−1^. It is believed that the PDMS solution of higher concentration would plug more metal aerogel pores, which reduces the conductive paths during compression (Figure S7, Supporting Information). Hence, we reveal that although PDMS reduces the sensitivity of flexible pressure sensors, it helps to improve their stability and reduce the effects of ambient humidity.

To understand the effect of the ratio of silver and gold on the sensing behaviour of the flexible pressure sensors, the sensing performances of Ag_X_Au_Y_ AG/MS with different ratios of silver to gold have been studied (*X*, *Y* represent the volume ratio of Ag and Au nanoparticles solution). As shown in the Figure S8 (Supporting Information), as the content of silver increases, the size of the aerogel ligaments becomes more nonuniform, and the amount of metal aerogels attached to the surface of the sponge skeleton also decreases. However, it is noted that the ratio of silver and gold basically will not significantly change the sensitivity of the sensors (Figure 4d). This can be attributed to the fact that the metal aerogels attached to the surface of the sponge skeleton will fall off when compressed (Figure 2b), and thus cannot provide more conductive channels during the compression process to change the resistance.

### 2.5 Applications of the Ag_2_Au_3_ AG/MS/PDMS_op_ Flexible Pressure Sensor

**Figure 5:**
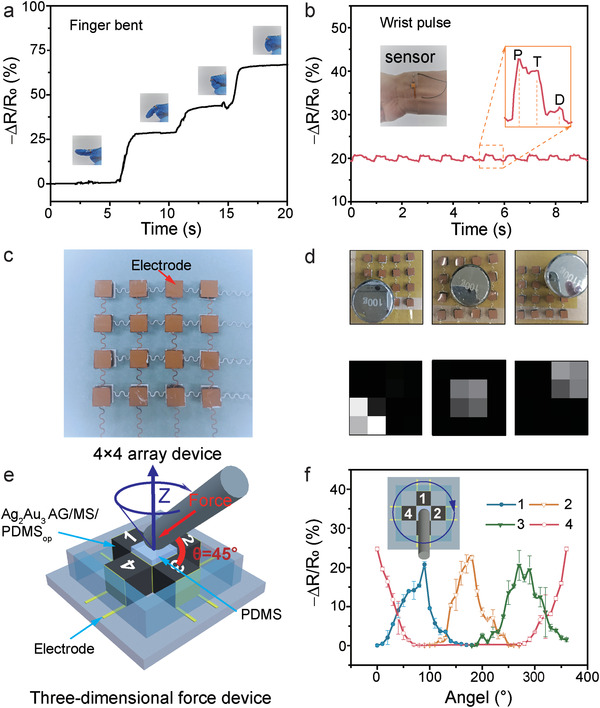
Application of the flexible pressure sensors in a) finger bending, b) pulse monitoring, c) 4 × 4 array device and d) the identification map of e) the location, and f) 3D force device. The scheme of e) 3D force device and f) the curves of the resistance of the four sensing units as a function of the force angle.

The Ag_2_Au_3_ AG/MS/PDMS_op_ flexible pressure sensor with excellent comprehensive performance is suitable for human state monitoring and real‐time health monitoring.^[^ ^29^ ^]^ The state change of the human body mainly depends on the rotation of various joints of the whole body, so the state of the human body can be obtained by detecting the state of the joints (Figure S9, Supporting Information). Fingers are the most flexible parts of human body. **Figure** **5**a shows that when the finger is in four different bending states, the resistance of the flexible pressure sensor also changes accordingly. To test the wrist pulse that can provide a lot of important information about human health, the flexible pressure sensor is tightly attached to the wrist artery. As shown in the Figure 5b, the real‐time record of the flexible sensor to the pulse signals corresponding heart rates were 78 beats min^−1^. The typical pulse signal shows three wave peaks, including percussion wave (P), tidal wave (T), and diastolic wave (D), all of them can be clearly identified, which reveals the potential of the flexible pressure sensor for real‐time health monitoring.^[^ ^3^, ^30^ ^]^

Beside the above‐mentioned application of the Ag_2_Au_3_ AG/MS/PDMS_op_ for a single site pressure sensing, it can be fabricated into 2D array device for providing pressure distribution information, which is very necessary in practical applications.^[^ ^31^ ^]^ Various shapes of Ag_2_Au_3_ AG/MS/PDMS_op_ sensing materials can be prepared easily by cutting the as prepared flexible Ag_2_Au_3_ AG/MS/PDMS_op_ or by preparing Ag_2_Au_3_ AG/MS/PDMS_op_ using melamine sponge precursor with different shapes (Figure S10, Supporting Information). The flexible pressure sensor with four pixels by four pixels array was fabricated as shown in Figure 5c. Each pixel has an area of 5 mm × 5 mm × 5 mm, and the pixels are connected by copper electrodes. Pressure sensor array can respond well to pressure and position, the weights are placed in different positions of the array sensor, and the positions of the weights can be displayed correspondingly, as shown in Figure 5d.

The application of the flexible pressure sensor is further extended to 3D force detection (Figure 5e). In the Ag_2_Au_3_ AG/MS/PDMS_op_ based 3D force device, four same sensing sites are distributed in four different directions, with a bendable post in the middle (Figure S11a,b, Supporting Information). As the force is applied to the sensing sites 1, 2, 3, and 4, resistance of each site changes (Figure S11c–f, Supporting Information). To verify the capability of the 3D force sensor device, a force of 8.8 N was applied along the direction with an angle of 45 degrees to the *X*–*Y* plane, tests were carried out every 10 degrees around the *Z*‐axis, the resistance values were collected and plotted, as shown in Figure 5f. As the rotation angle changes, the resistance of the corresponding sensing site changes sinusoidally, indicating that the 3D force sensor device has good performance. The high performances of the above two devices demonstrate the potential of the Ag_2_Au_3_ AG/MS/PDMS_op_ flexible pressure sensor in electronic skins.

## 3 Conclusions

In conclusion, a facile “interfacial locking strategy” has been developed to fabricate flexible pressure sensors with excellent comprehensive performances including high sensitivity and especially outstanding durability. Benefiting from the synergy of the stable elasticity of the melamine sponge, the interfacial locking effect of the thin PDMS layer on the sponge skeletons and the metal aerogels, and the hierarchically porous structure of the metal aerogels, the as prepared Ag_2_Au_3_ AG/MS/PDMS_op_ flexible pressure sensor exhibits sensitivity as high as 12 kPa^−1^, a response time as fast as 85 ms, and excellent durability over 23 000 compression cycles. The excellent performance enables the sensor's successful applications not only in real‐time monitoring of finger motions and wrist pulse, but also as a 2D array device for pressure mapping and as a 3D force‐detecting device. This work opens a new avenue for the development of piezoresistive sensors with both high sensitivity and long‐term durability. The approach proposed in this study also breaks the bottleneck of the intrinsically poor mechanical properties of metal aerogels and widens their applications in a variety of fields including electronic skin, wearable devices, and HMI.

## Conflict of Interest

The authors declare no conflict of interest.

## Supporting information

Supporting Information

Supplemental Video 1

## Data Availability

Research data are not shared.
