# Supplementary material for: Interfacially Locked Metal Aerogel Inside Porous Polymer Composite for Sensitive and Durable Flexible Piezoresistive Sensors
Source: Adv Sci (Weinh). 2022 Jun 24;9(23):2201912. doi: 10.1002/advs.202201912 (PMC9376829; doi:10.1002/advs.202201912)
Supplement: Supplementary file 1 — Supporting Information [file ADVS-9-2201912-s002.pdf]

ADVANCED SCIENCE
Open Access

# Supporting Information

for *Adv. Sci.*, DOI 10.1002/advs.202201912

Interfacially Locked Metal Aerogel Inside Porous Polymer Composite for Sensitive and Durable Flexible Piezoresistive Sensors

*Jian Li, Ning Li, Yuanyuan Zheng, Dongyang Lou, Yue Jiang, Jiaxi Jiang, Qunhui Xu, Jing Yang, Yujing Sun, Chuxuan Pan, Jianlan Wang, Zhengchun Peng*, Zhikun Zheng* and Wei Liu**

# Supporting Information

**Interfacially Locked Metal Aerogel Inside Porous Polymer Composite for Sensitive and Durable Flexible Piezoresistive Sensors**

*Jian Li, Ning Li, Yuanyuan Zheng, Dongyang Lou, Yue Jiang, Jiaxi Jiang, Qunhui Xu, Jing Yang, Yunjing Sun, Chuxuan Pan, Jianlan Wang, Zhengchun Peng,* Zhikun Zheng,* Wei Liu**

## Experimental Section

**Materials**: Silver nitrate ($AgNO_3$, 99.9+%), Trisodium citrate (anhydrous, 99%) were purchased from Alfa Aesar, Gold chloride trihydrate ($HAuCl_4$-$3H_2O$, ≥99.9%), Potassium tetrachloropalladate (II) ($K_2PdCl_4$) (99.99%), Chloroplatinic acid hydrate ($H_2PtCl_6$) (≥ 99.995%), Potassium tetrachloroplatinate (II) ($K_2PtCl_4$) (99.9%), Sodium borohydride ($NaBH_4$, 98%), n-Hexane (>99%), PVP ($M_W \approx 37\,000$), glycerol, were purchased from Aladdin. Melamine Sponge was purchased from Shanghai Chuangyoujiancai. SYLGARD™ 184 Silicone Elastomer Base and SYLGARD™ 184 Silicone Elastomer Curing Agent were purchased from Dow Corning, America. Petroleum ether 60-90 was purchased from Guangdong Guanghua Sci-Tech Co., Ltd. Dimethylsilicone oil (viscosity: 1000 mPa.s, 250 °C) was purchased from Shanghai Titan Scientific Co., Ltd. Ethanol was obtained from Guangzhou Chemical Reagent Factory. Sulfuric acid (98%, GR) and hydrochloric acid (HCl, 36~38%, GR) were purchased from Chengdu Chron Chemicals used without any treatment. All the chemicals were used as received without any further treatment. The deionized water used in the experiment was purified by the Milli-Q instrument.

**Preparation of silver nanoparticles solution (Ag NPs)**: Ag NPs were synthesized according to the procedure described in the literature.[1] In a typical synthesis, 0.14 mmol of $AgNO_3$ was added to 500 mL of boiling deionized water. After stirring for 10 min, 11.6 mL 1% (w) trisodium citrate solution was added quickly into the $AgNO_3$ solution. 1.5 minutes later, 5.5

mL of trisodium citrate (1 wt%) and sodium borohydride (0.076 wt%) mixed solution was quickly added. After 30 minutes, the suspension was taken out and stored in the dark to cool to room temperature.

**Preparation of gold nanoparticles solution (Au NPs)**: Au NPs were synthesized according to the procedure described in the literature.[1] In a typical synthesis, 0.14 mmol of $HAuCl_4$ was added to 500 mL of deionized water. After stirring for 10 min, 11.6 mL 1 wt% trisodium citrate solution was added into $HAuCl_4$ solution quickly. 1.5 minutes later, 5.8 mL trisodium citrate (1 wt%) and sodium borohydride (0.17 wt%) mixed solution was quickly added. After 30 minutes, the suspension was taken out and stored in the dark.

**Preparation of concentrated nanoparticles solution**: All the obtained NPs solution was then concentrated via centrifuge filters (Sartorius, Vivaspin, 20 mL, MWCO 30000). The centrifuge filters were washed three times with water before use. First, the NPs solution was concentrated to 15 mL in a step-wise manner, and diluted with water to 120 mL, then re-concentrated to 15 mL using a centrifugal filter. This step was repeated for 5 times. Finally, the NPs solution was concentrated to 5 mL, and the highly concentrated nanoparticles solution was obtained.

**Preparation of Ag NWs**: Ag NWs were synthesized according to the procedure described in the reference.[2] 1 g PVP ($M_W \approx 37\,000$) was added into 40 mL glycerol and heated in an oil bath at 60 °C for 30 minutes under magnetic stirring. Then, the reaction mixture was transferred into an ice water bath until the temperature of the solution decreased to 40 °C. Then, 0.3 mL (0.5 g) of aqueous $AgNO_3$ and 0.2 mL (30 mg) of aqueous NaCl solution were injected under continuous stirring, and the reaction mixture was transferred to a 50 mL autoclave and heated in a 180 °C oven for 16 h. Upon completion of the reaction, the autoclave was removed from the oven and cooled to room temperature. After sedimentation for 48 h, the upper suspension was poured out. Ag NWs suspensions were obtained with a concentration of 20 mg $mL^{-1}$.

**Preparation of Pt, Pd, PtPd aerogel**: Pt, Pd, PtPd aerogel were synthesized according to the procedure described in the reference.[3]

**Preparation of $Ag_2Au_3$ AG/MS/$PDMS_{op}$**: Firstly, 50 μL of concentrated Ag NPs solution and 75 μL of concentrated Au NPs solution were mixed well. Then, 5*5*5 mm melamine sponge

was used to absorb the mixed nanoparticles solution. After that, melamine sponge was put into dimethylsilicone oil and heated in an oven for 12 hours to make the gold particles and silver particles form hydrogel in the melamine sponge. Then, the resulted sponge was taken out and soaked in petroleum ether for 2 h to wash off the dimethylsilicone oil. After exchanging with ethanol and supercritical drying, $Ag_2Au_3$ AG/MS was obtained. Then, 70 mg of a mixture of SYLGARD™ 184 Silicone Elastomer Base and SYLGARD™ 184 Silicone Elastomer Curing Agent (mass ratio 10:1) was added into 30 mL of n-hexane. Finally, the $Ag_2Au_3$ AG/MS was soaked in the mixed solution for two hours, taken out and put in the oven to heat at 75 degrees for 3 hours to obtain the final $Ag_2Au_3$ AG/MS/$PDMS_{op}$.

**Preparation of Ag NW/MS /$PDMS_{op}$**: Here, the preparation of Ag NW /MS/$PDMS_{op}$ is described. A 5*5*5 mm melamine sponge was used to absorb 125 μL Ag NW solution, then was put in an oven at 75 °C for 12 h to obtain Ag NW/MS. After that, 70 mg of a mixture of SYLGARD™ 184 Silicone Elastomer Base and SYLGARD™ 184 Silicone Elastomer Curing Agent (mass ratio 10:1) was added to 30 mL of n-hexane. Then, the Ag NW/MS was soaked in for two hours, taken out and put in the oven to heat at 75 °C for 3 hours to obtain Ag NW/MS/$PDMS_{op}$.

**Preparation of $Ag_2Au_3$ AG/MS, $Ag_2Au_3$ AG/MS/$PDMS_{op}$, Ag NW/MS, Ag NW/MS/$PDMS_{op}$ flexible pressure sensor**: Usually, after pre-preparing a 5*5*5 mm $Ag_2Au_3$ AG/MS, $Ag_2Au_3$ AG/MS/$PDMS_{op}$, Ag NW/MS, or Ag NW/MS/$PDMS_{op}$, conductive silver paste was used as a binder to install the upper and lower copper electrodes and solder the wires to obtain a flexible pressure sensor.

**Characterizations**: Scanning electron microscopy (SEM) and energy dispersive spectroscopy (EDS) were performed using a field-emission scanning electron microscope (TESCAN CLARA). Transmission electron microscopy (TEM) and TEM-EDS were carried out on a FEI Tecnai T12 transmission electron microscope operated at 120 kV. X-ray diffraction (XRD) was conducted on a Rigaku SmartLab X-ray diffractometer operated at 40 kV and 40 mA with Cu-Kα radiation ($\lambda$=1.5405 Å). Attenuated total reflection Fourier transformed infrared (ATR-FTIR) spectra were measured on a Thermo Scientific Nicolet iS10. Performance of the proximity sensor was characterized using a dynamic fatigue testing system (ElectroPuls 1000,

Instron). the real-time resistance change of the sensor was recorded using a digital multimeter (Keithley 6500). Current−voltage (I−V) curves of the sensors were measured with a source meter (Keithley 2400).

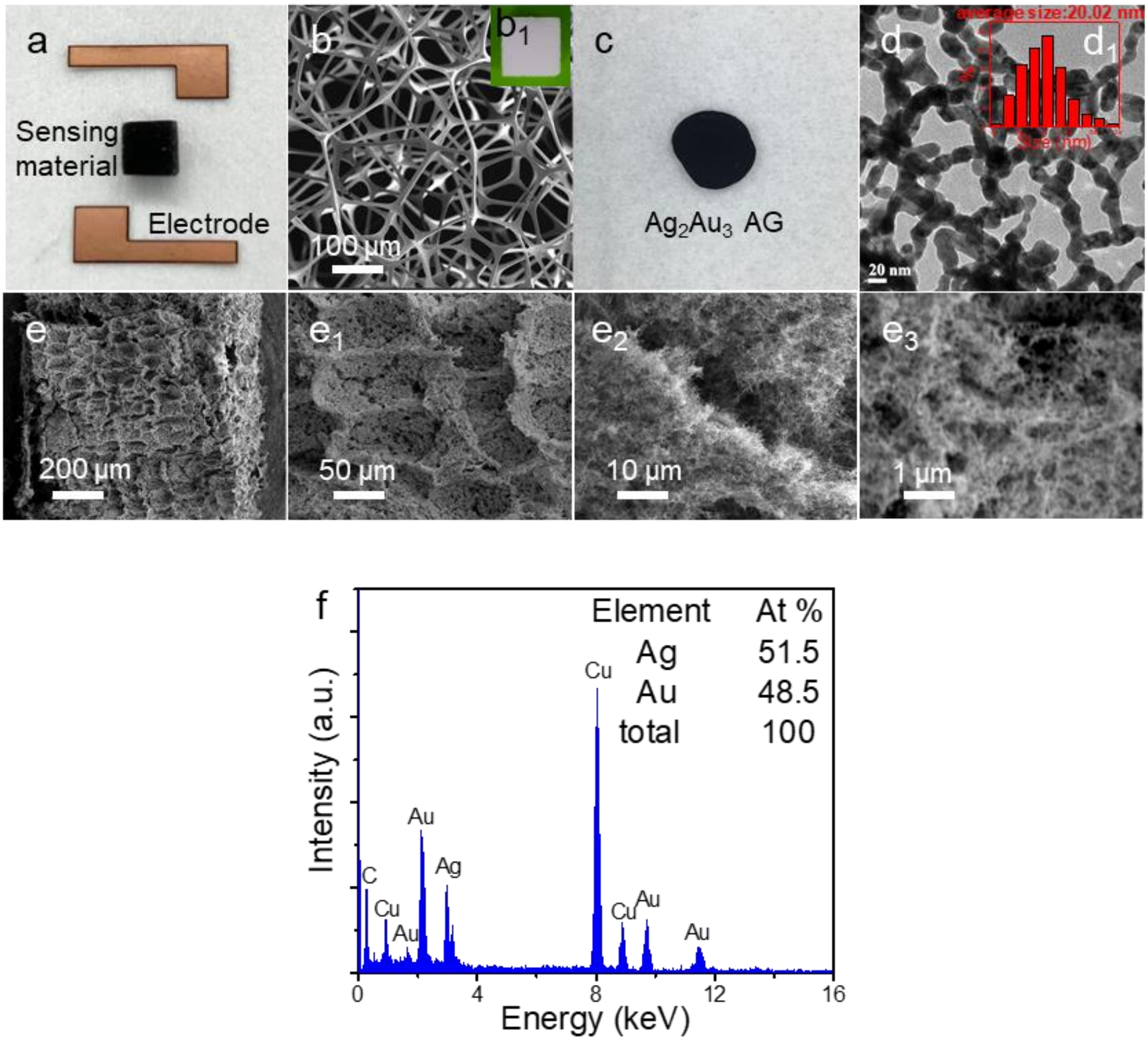


**Figure S1.** (a) Photograph of components of the flexible sensor. (b) The SEM images of melamine sponge and ($b_1$) photograph of melamine sponge. (c) Photograph of $Ag_2Au_3$ AG. (d) The TEM image of the $Ag_2Au_3$ AG and ($d_1$) the size distribution of ligaments. (e, $e_1$, $e_2$, $e_3$) The SEM images of the $Ag_2Au_3$ AG. The surface of the pothole about one hundred microns wide was caused by the laser cutting. (f) TEM-EDS of the $Ag_2Au_3$ AG.

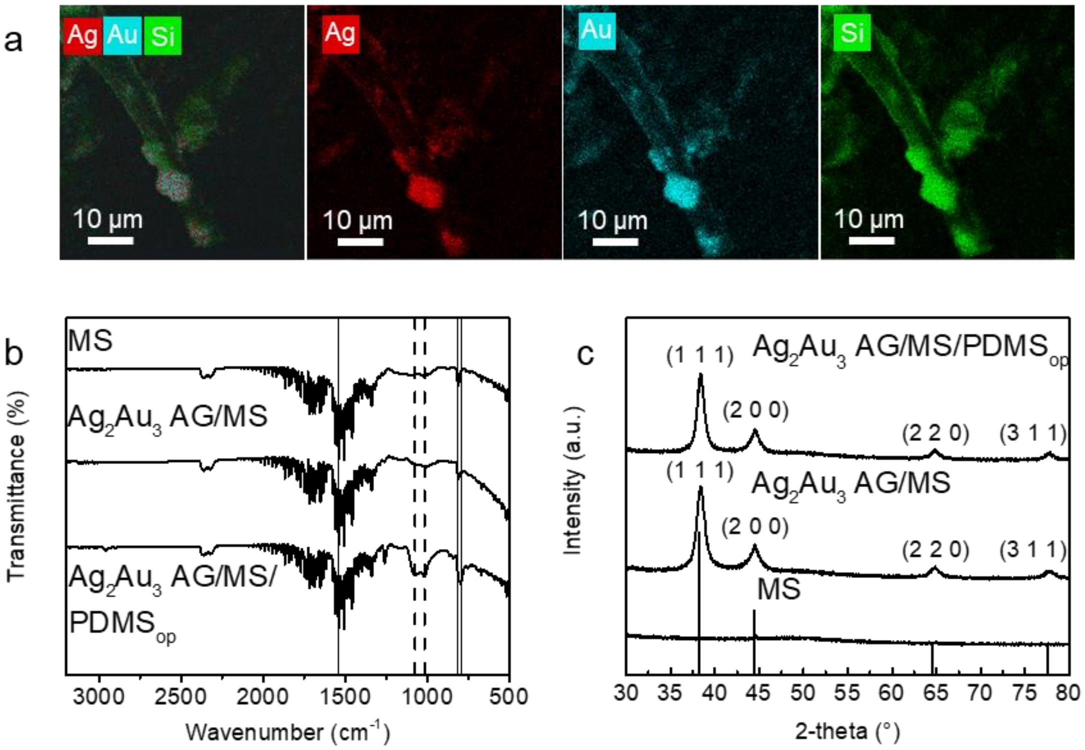


**Figure S2.** (a) The SEM-EDS mapping of the $Ag_2Au_3$ AG/MS/$PDMS_{op}$. (b) ATR-IR of the melamine sponge, $Ag_2Au_3$ AG/MS and $Ag_2Au_3$ AG/MS/$PDMS_{op}$. (c) XRD of the melamine sponge, $Ag_2Au_3$ AG/MS and $Ag_2Au_3$ AG/MS/$PDMS_{op}$.

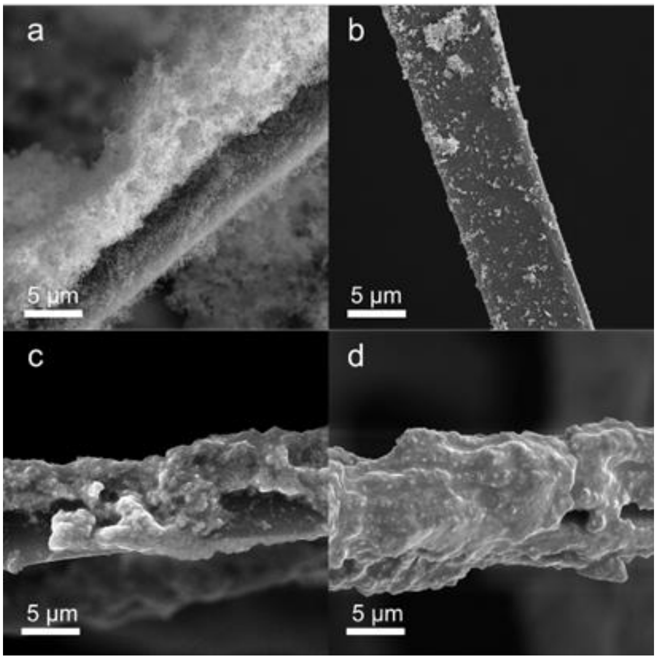


**Figure S3.** SEM images of the $Ag_2Au_3$ AG/MS (a) before and (b) after repeated loading experiments. SEM images of the $Ag_2Au_3$ AG/MS/$PDMS_{op}$ (c) before and (d) after repeated loading experiments.

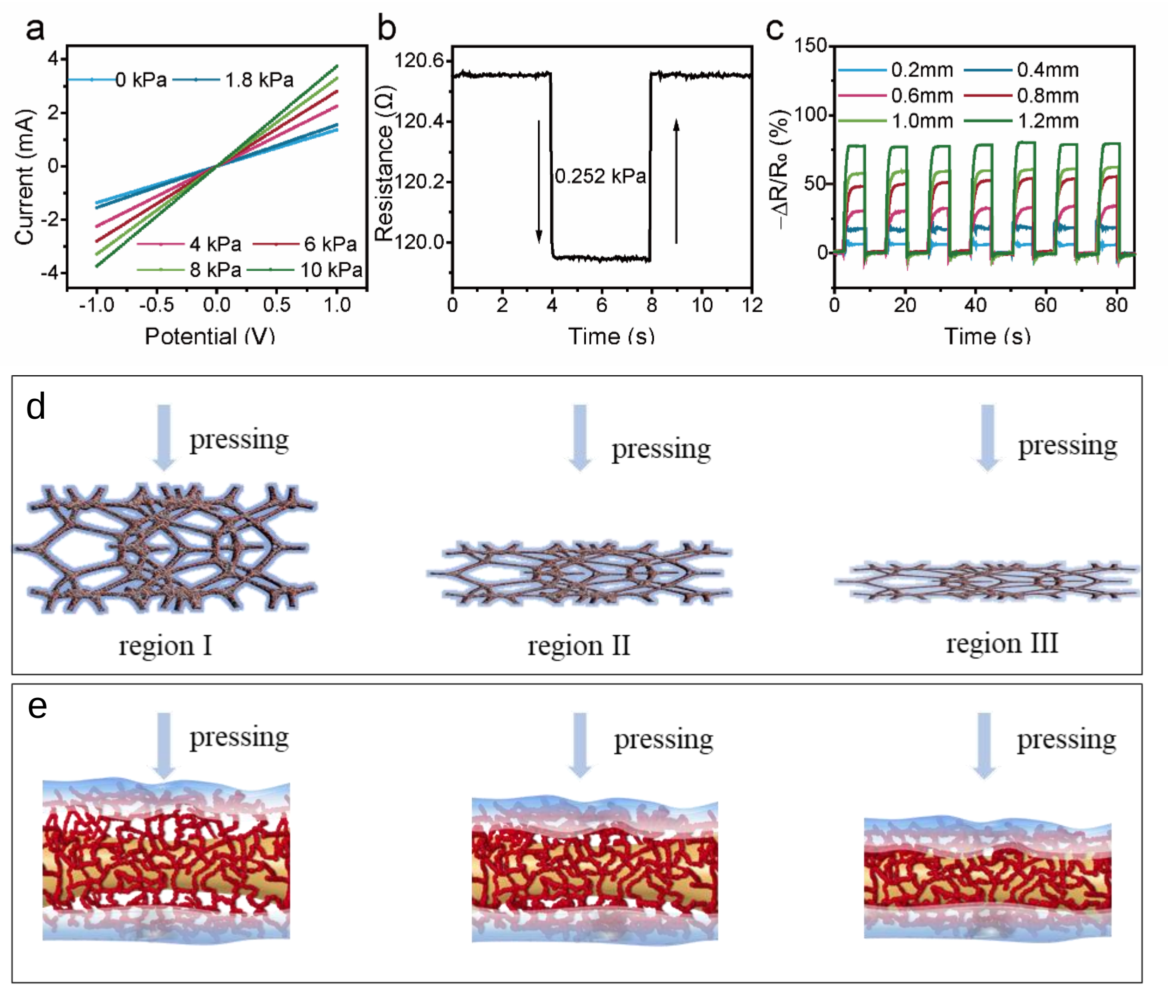


**Figure S4.** (a) The I-V curves of the $Ag_2Au_3$ AG/MS/PDMS$_{op}$ flexible sensor at different pressure. (b) Resistance response of the pressure sensor at 0.252 kPa. (c) The response of square wave signals at different compression displacement. (d) Schematic illustration of the $Ag_2Au_3$ AG/MS/PDMS$_{op}$ flexible sensor deformation during compression. (e) Proposed evolution of metal aerogel under pressure in the $Ag_2Au_3$ AG/MS/PDMS$_{op}$ composite.

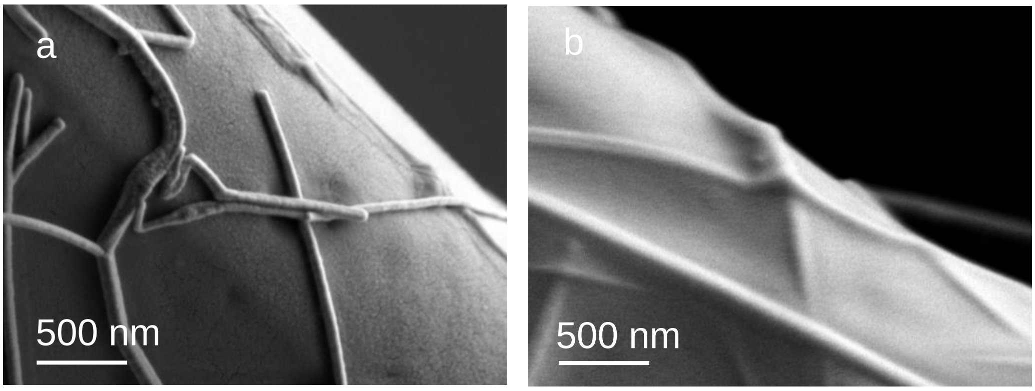


**Figure S5.** The SEM images of (a) Ag NW/MS and (b) Ag NW/MS/PDMS.

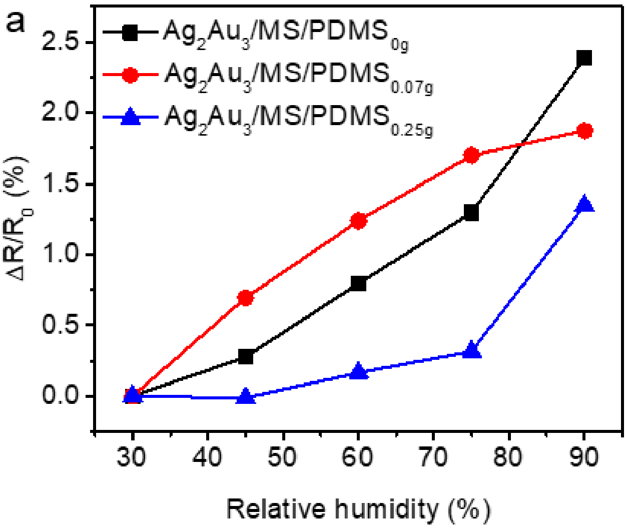


**Figure S6.** The effect of humidity on resistance at 25°C. The humidity ranges from 30% to 90%.

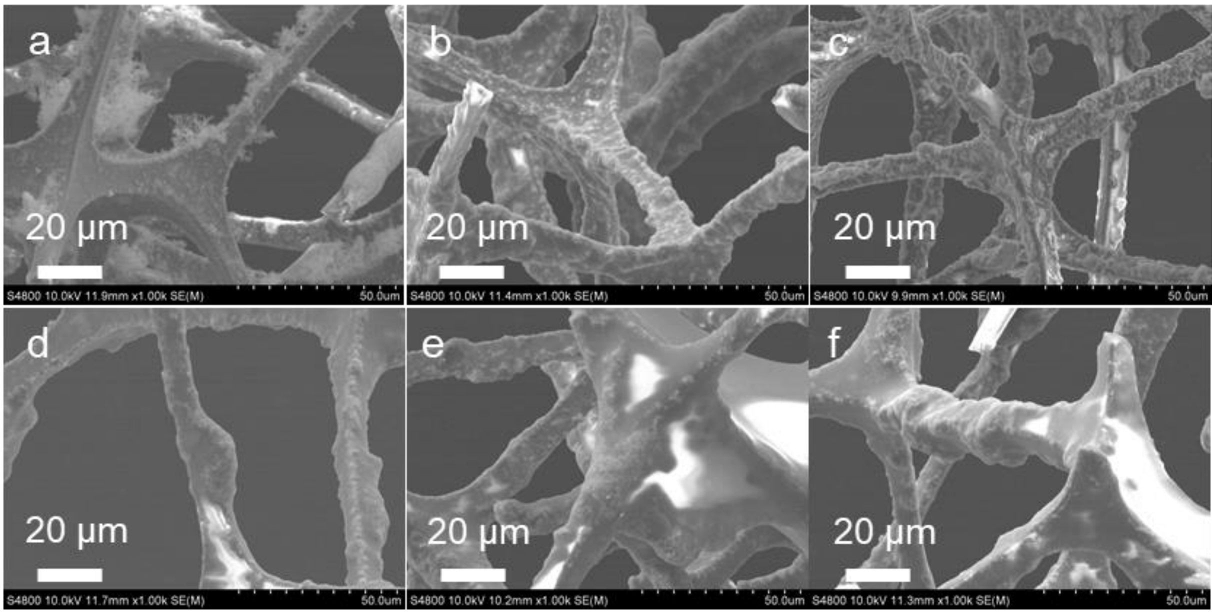


**Figure S7.** The SEM images of different PDMS contents. (a) 0.000 g, (b) 0.070 g, (c) 0.125 g, (d) 0.250 g, (e) 0.500 g, (f) 1.000 g.

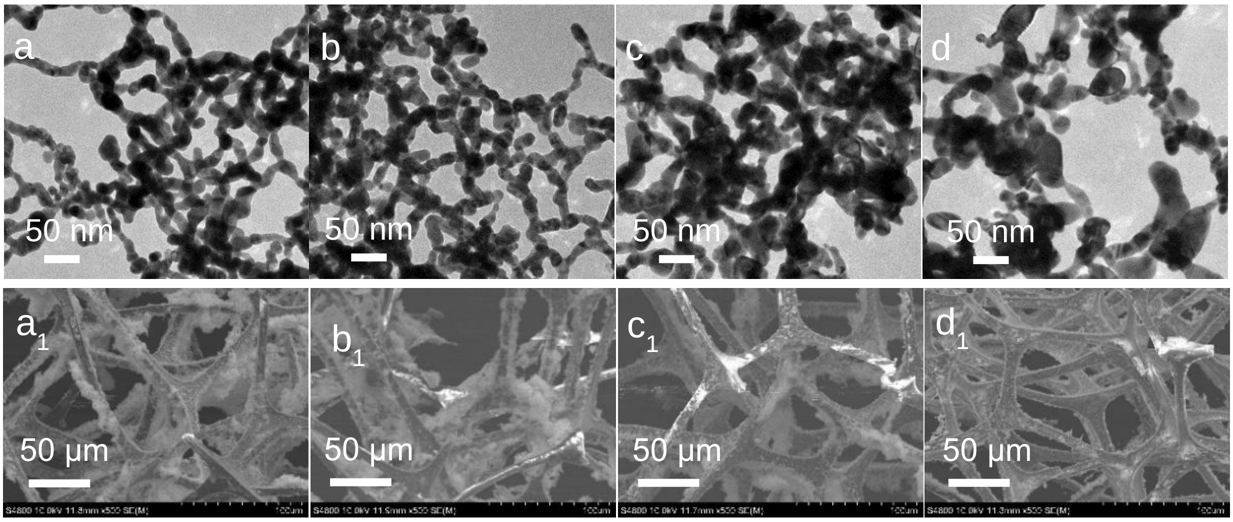


**Figure S8.** The effect of gold and silver ratio on performance. The TEM images of $Ag_XAu_Y$ AG/MS with different silver and gold ratio (a) 1:4, (b) 2:3, (c) 3:2, (d) 4:1. The corresponding SEM images ($a_1$), ($b_1$), ($c_1$), ($d_1$).

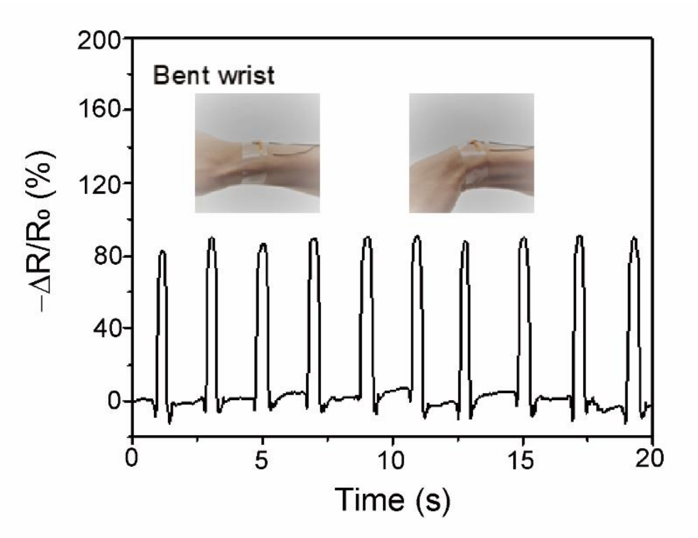


**Figure S9.** The ($R_0$-R)/$R_0$-Pressure curve of the response of $Ag_2Au_3$ AG/MS/$PDMS_{op}$ to bent wrist.

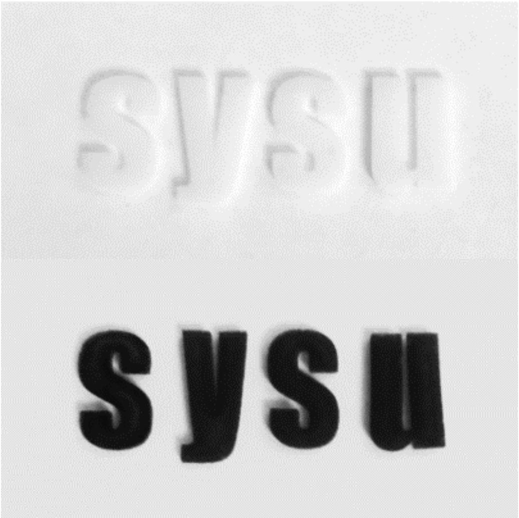


**Figure S10.** Different shapes of melamine sponges (white) and $Ag_2Au_3$ AG/MS/$PDMS_{op}$ (black).

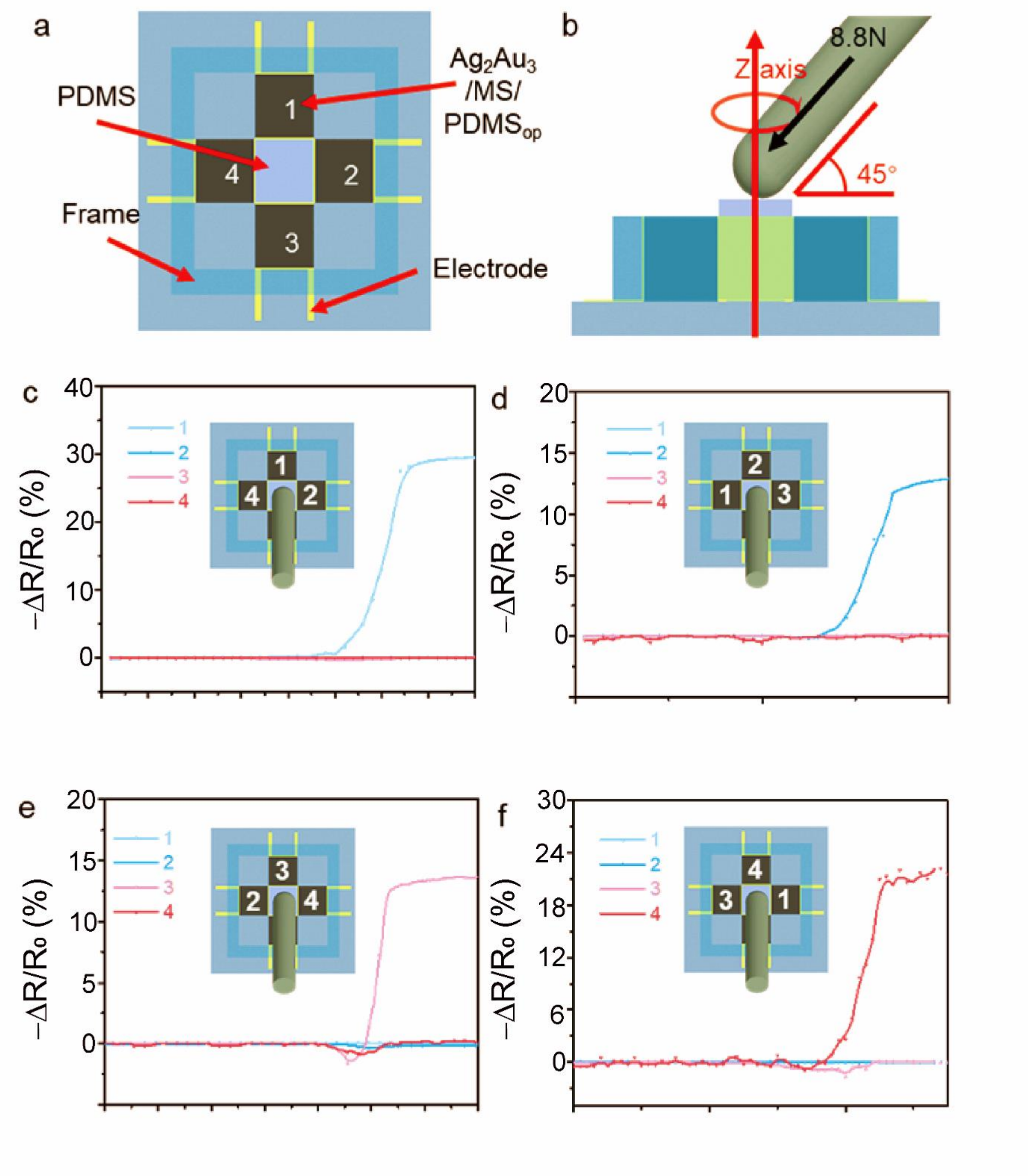


**Figure S11.** Three-dimensional force devices and performance. (a) Top view and (b) side view of 3D force device. (c-f) are the resistance response curve of sensor materials 1, 2, 3, and 4 when the angle is 45° and force is 8.8 N.

**Table S1.** Summary of the performances of flexible metal-based piezoresistive pressure sensors reported.

| Sensing material | Transduction principle | Highest sensitivity and region | Response time / recovery time | Stability (cycle) | Application | Reference |
|---|---|---|---|---|---|---|
| Au NWs/ polyacrylamide | piezoresistive | △$I/I_0$=3.71 $kPa^{-1}$ <2.8 kPa | 500 ms/ 590 ms | 10000 | E-hand skin | [4] |
| Au NWs impregnated tissue paper | piezoresistive | △$I/I_0$=1.14 $kPa^{-1}$ <5.5 kPa | <17 ms | >50000 | in-situ real-world force signals from radial artery blood pulses and acoustic vibrations | [5] |
| Au sputtered on PU sponge | piezoresistive | △$R/R_0$=0.059 $kPa^{-1}$ <4.7 kPa | 9 ms | 1000 | small-/large-scale motion monitoring | [6] |
| AuAg sea urchin shaped nanoparticles/PU film | piezoresistive | △$R/R_0$=2.46 $kPa^{-1}$ <1 kPa | 30 ms | 200 | distribution of finger-pressure sensing arrays | [7] |
| Ag-PPy aerogel | piezoresistive | / | 230 ms/ 190 ms | 1000 | / | [8] |
| Ag NWs/ $Ti_3C_2Tx$ MXene aerogel | piezoresistive | △$I/I_0$=645.69 $kPa^{-1}$ <1 kPa | 60 ms/ 114 ms | 1000 | human motion monitoring | [9] |
| PPy-Ag NW aerogel-sponge | piezoresistive | △$R/R_0$=0.33 $kPa^{-1}$ <3 kPa | <1 ms | / | / | [10] |
| Ag NW/ PU films | piezoresistive | △$R/R_0$=625.8 $kPa^{-1}$ <40 pa | 20 ms/ 30 ms | >2300 | monitor various motions | [11] |
| natural rubber latex foam/ polydopamine/Ag NP | piezoresistive | GF=-81 <40 % | / | 1000 | human movement monitoring | [12] |

| | | | | | | |
|---|---|---|---|---|---|---|
| Ag NWs/POE nanofiber | piezoresistive | $\triangle I/I_0$=19.4 kPa$^{-1}$ <2.76 kPa | 30 ms/ 42 ms | >6000 | detecting the spatial pressure distribution and monitoring human muscle motions | [13] |
| Ag NWs@ carbonized melamine foam | piezoresistive | $\triangle I/I_0$=4.97 kPa$^{-1}$ 30–50 kPa | 300 ms/ 200 ms | 1000 | monitor tiny movements in the human body | [14] |
| Ag NWs-PDMS composite | piezoresistive | $\triangle R/R_0$=0.11 kPa$^{-1}$ <1.5 kPa | 60 ms/ 20 ms | 2000 | human motion tracking, physiological signal monitoring | [15] |
| Ag NWs coated on porous PDMS | piezoresistive | $\triangle R/R_0$=14.1 kPa$^{-1}$ <3.5 kPa | 47 ms | 1000 | Arrays detect physiological signals (pulse, voice, motion movement) | [16] |
| Cu NW based aerogel | piezoresistive | $\triangle I/I_0$=0.7 kPa$^{-1}$ <0.21 kPa | ~80 ms | 200 | / | [17] |
| Cu NW-PVA aerogel | piezoresistive | $\triangle R/R_0$=0.036 kPa$^{-1}$ <4 kPa | / | 10000 | / | [18] |
| Cu NPs/nylon | piezoresistive | $\triangle I/I_0$=0.57 kPa$^{-1}$ | 2 ms | 6000 | detect subtle stress or movement for healthcare monitoring. | [19] |
| Cu NWs-PVA | piezoresistive | $\triangle I/I_0$=0.267 kPa$^{-1}$ <10 kPa | / | / | / | [20] |
| Cu NWs-PVA-Ecoflex | piezoresistive | $\triangle R/R_0$=0.159 kPa$^{-1}$ <6 kPa | / | 10000 | arrays | [21] |
| C-PDMS/ Ag NW/PU | piezoresistive | $\triangle I/I_0$=264 kPa$^{-1}$ 4-10 kPa | 80 ms/ 60 ms | 4000 | arrays | [22] |
| $WS_2$-nanosheet-wrapped sponges | piezoresistive | $\triangle I/I_0$=0.39 kPa$^{-1}$ <125 Pa | / | 10000 | underwater detection of tiny vibration | [23] |

| | | | | | | |
|---|---|---|---|---|---|---|
| CA@PU Sponge | piezoresistive | GF=26.07 <0.6 % | / | 500 | human motion detection, arrays | [24] |
| $Fe_2O_3$/C@ $SnO_2$ sponge | piezoresistive | $\triangle I/I_0$=680 $kPa^{-1}$ <10 kPa | 10 ms/ 22 ms | 3500 | wearable electronics, health monitoring, and measuring pressure distribution | [25] |
| **AgAu AG /MS/ PDMS** | **piezoresistive** | **$\triangle R/R_0$=12 $kPa^{-1}$ 3-12 kPa** | **85 ms** | **>23000** | **human motions and health monitoring, 3D force detection, arrays** | **This work** |

# References


[1] A. K. Herrmann, P. Formanek, L. Borchardt, M. Klose, L. Giebeler, J. Eckert, S. Kaskel,

N. Gaponik, A. Eychmüller, *Chem. Mater.* **2014**, *26*, 1074.

[2] M. Hassan, G. Abbas, Y. Lu, Z. Wang, Z. Peng, *J. Mater. Chem. A* **2022**, *10*, 4870.

[3] W. Liu, P. Rodriguez, L. Borchardt, A. Foelske, J. Yuan, A. K. Herrmann, D. Geiger, Z. Zheng, S. Kaskel, N. Gaponik, R. Kötz, T. J. Schmidt, A. Eychmüller, *Angew. Chem. Int. Ed*. **2013**, *52*, 9849-9852.

[4] M. J. Yin, Y. Zhang, Z. Yin, Q. Zheng, A. P. Zhang, *Adv. Mater. Technol.* **2018**, *3*, 1800051.

[5] S. Gong, W. Schwalb, Y. Wang, Y. Chen, Y. Tang, J. Si, B. Shirinzadeh, W. Cheng, *Nat. Commun.* **2014**, *5*, 3132.

[6] Y. H. Wu, H. Z. Liu, S. Chen, X. C. Dong, P. P. Wang, S. Q. Liu, Y. Lin, Y. Wei, L. Liu, *ACS Appl. Mater. Interfaces* **2017**, *9*, 20098.

[7] D. Lee, H. Lee, Y. Jeong, Y. Ahn, G. Nam, Y. Lee, *Adv. Mater.* **2016**, *28*, 9364.

[8] B. Liang, J. Wu, Q. Feng, J. Huang, T. Zhang, Z. Yan, *Mater. Lett.* **2020**, *279*, 128474.

[9] L. Bi, Z. Yang, L. Chen, Z. Wu, C. Ye, *J. Mater. Chem. A* **2020**, *8*, 20030.

[10] W. He, G. Li, S. Zhang, Y. Wei, J. Wang, Q. Li, X. Zhang, *ACS Nano* **2015**, *9*, 4244.

[11] G. J. Zhu, P. G. Ren, J. Wang, Q. Duan, F. Ren, W. M. Xia, D. X. Yan, *ACS Appl. Mater. Interfaces* **2020**, *12*, 19988.

[12] W. Zhang, L. Lin, L. Zhang, Y. Wang, Y. Zhuang, Y. Choi, Y. Cho, T. Chen, H. Yao, Y. Piao, *ACS Appl. Polym. Mater.* **2021**, *4*, 54.

[13] W. Zhong, C. Liu, Q. Liu, L. Piao, H. Jiang, W. Wang, K. Liu, M. Li, G. Sun, D. Wang, *ACS Appl. Mater. Interfaces* **2018**, *10*, 42706.

[14] B. Cai, L. Wang, F. Yu, J. Jia, J. Li, X. Li, X. Yang, Y. Jiang, W. Lü, *Applied Physics A* **2021**, *128*, 6.

[15] Y. Li, D. Han, C. Jiang, E. Xie, W. Han, *Adv. Mater. Technol.* **2019**, *4*, 1800504.

[16] L. Dan, S. Shi, H. J. Chung, A. Elias, *ACS Appl. Nano Mater.* **2019**, *2*, 4869.

[17] X. Xu, R. Wang, P. Nie, Y. Cheng, X. Lu, L. Shi, J. Sun, *ACS Appl. Mater. Interfaces* **2017**, *9*, 14273.

[18] Y. Tang, S. Gong, Y. Chen, L. W. Yap, W. Cheng, *ACS Nano* **2014**, *8*, 5707.

[19] J. Chen, J. Zhang, J. Hu, N. Luo, F. Sun, H. Venkatesan, N. Zhao, Y. Zhang, *Adv. Mater.* **2022**, *34*, 2104313.

[20] J. Huang, H. Wang, B. Liang, U. J. Etim, Y. Liu, Y. Li, Z. Yan, *Chem. Eng. J.* **2019**, *364*, 28.

[21] L. W. Yap, S. Gong, Y. Tang, Y. Zhu, W. Cheng, *Science Bulletin* **2016**, *61*, 1624.

[22] J. H. Lee, J. S. Heo, Y. J. Kim, J. Eom, H. J. Jung, J. W. Kim, I. Kim, H. H. Park, H. S. Mo, Y. H. Kim, S. K. Park, *Adv. Mater.* **2020**, *32*, 2000969.

[23] R. Xu, K. Zhang, X. Xu, M. He, F. Lu, B. Su, *Adv. Sci.* **2018**, *5*, 1700655.

[24] S. Zhang, H. Liu, S. Yang, X. Shi, D. Zhang, C. Shan, L. Mi, C. Liu, C. Shen, Z. Guo, *ACS Appl. Mater. Interfaces* **2019**, *11*, 10922.

[25] X. Wang, L. Tao, M. Yuan, Z. Wang, J. Yu, D. Xie, F. Luo, X. Chen, C. Wong, *Nat. Commun.* **2021**, *12*, 1776.
